# Energetics, kinematics, and physiologic aspects of tango walking in the leader role

**DOI:** 10.7717/peerj.21581

**Published:** 2026-08-06

**Authors:** Carol Torres, Alberto E. Minetti, Carlo M. Biancardi, Gabriel Fábrica

**Affiliations:** 1 Departamento de Educación Física y Deporte, Instituto Superior de Educación Física, Universidad de la República, Montevideo, Montevideo, Uruguay; 2 Laboratory of Locomotion Physiomechanics, Department of Pathophysiology and Transplantation, University of Milan, Milan, Lombardy, Italy; 3 LIBiAM, Department of Biological Sciences, CENUR Litoral Norte–Universidad de la República, Paysandú, Paysandú Department, Uruguay; 4 Unidad Académica de Biofísica, Facultad de Medicina, Universidad de la República, Montevideo, Montevideo, Uruguay; 5 Department of Sports Sciences and Physical Conditioning, Education Faculty, Universidad Católica de la Santísima Concepción, Concepción, Bio Bio, Chile

**Keywords:** Walking energetics, Metabolic cost, Mechanical work of locomotion, Gait biomechanics, Center of mass trajectory, Dance-based rehabilitation, Gait variability, Locomotor efficiency, Dance-based walking

## Abstract

**Background:**

Tango has therapeutic potential to improve gait and quality of life in populations with locomotor impairments. However, quantitative biomechanical and metabolic evidence remains limited. Tango walking in the leader role involves gait modifications that may affect locomotor economy. This study quantified metabolic cost, mechanical work, centre of mass motion and energetics during tango walking, and compared these variables with self-selected walking at similar speeds. We hypothesized that tango walking would increase cost of transport, reduce efficiency, and be associated with modifications in centre of mass motion and spatiotemporal gait parameters.

**Methods:**

Seventeen advanced tango dancers participated in the study, performing two 5-minute trials: self-selected walking and tango walking in the leader role just Milonga music. Oxygen consumption was measured to compute net metabolic power and cost of transport. Whole-body kinematics were recorded using a 3D motion capture system to reconstruct the centre of mass trajectories and compute spatiotemporal and mechanical variables. Statistical comparisons between conditions were performed using paired tests for discrete variables and Statistical Parametric Mapping (SPM) for time-series data.

**Results:**

Walking speed did not differ between conditions. Tango walking increased metabolic power and cost of transport (≈+50%) and reduced efficiency, without significant changes in mechanical work. SPM analyses revealed centre of mass trajectories with reduced lateral oscillations during mid-stance and greater knee flexion throughout stance.

**Conclusions:**

Tango walking to a milonga rhythm (stride frequency: 0.87 ± 0.22 Hz) increases metabolic demand without proportional changes in mechanical work. These changes are accompanied by alterations in knee kinematics and centre of mass trajectory. The findings suggest potential mechanisms underlying the therapeutic effects of tango on gait and balance, which require confirmation in neurological populations.

## Introduction

Tango was declared an Intangible Cultural Heritage of Humanity by United Nations Educational, Scientific and Cultural Organization (UNESCO) (2026), and based on historical analyses, first emerged as a dance form deeply linked to its musical structure (Gobello, 1999; Vidart, 2007). The term tango typically encompasses three musical subgenres: “tango”, “vals criollo”, and “milonga ciudadana”, whose association is grounded primarily in shared cultural and social contexts, as reflected in their regular alternation within a single milonga event. Beyond their artistic context, these dances have been used as therapeutic exercise to enhance balance, gait, and quality of life in individuals with locomotor impairments, including Parkinson’s disease (McKee & Hackney, 2013; Lötzke, Ostermann & Büssing, 2015; Rabinovich et al., 2021). In this sense, evidence suggests that such dances contribute to alleviating both physical symptoms (Rabinovich et al., 2021) and cognitive functioning (McKee & Hackney, 2013), as they incorporate low-level aerobic activity and movements that challenge gait and balance. In addition, tango dance practice has the advantage of requiring multitasking high-level and progressive learning of motor skills in the presence of external cues, for example those provided by the music (Hackney & Earhart, 2009; Hackney & Earhart, 2010; Lötzke, Ostermann & Büssing, 2015). However, mechanical and physiologic characteristics of traditional tango dance remain to be elucidated, despite the potential of such information to support clinical translation.

The essence for the three subgenres of tango dance is walking as one would walk down the street, but performed with cadence that is rhythmically cued by the music’s periodic pattern and physicality that is aesthetically stylized (Laguna, 2020). So, this dance is clearly defined by the action of walking, and one of the initial aims when commencing tango classes is to learn to walk in the tango style. Here the two roles emerge: the “leader”, which implies forward walking, and the “follower”, which implies backward walking. Tango walking in the leader role requires a forward shift of the body’s longitudinal axis, maintained continuously during the transition between steps while preserving gait symmetry. In addition, although specific stylistic variations exist across subgenres, tango walking is typically characterized, according to tango pedagogy, by greater pelvic rotation in the transverse plane and increased knee flexion during stance compared to normal walking, although this feature has not been quantitatively characterized across the gait cycle using objective biomechanical methods. These aspects may imply changes in the mechanics and economy of the gait (Saibene & Minetti, 2003), whose knowledge is relevant for the use of tango dance in different patient populations. However, existing biomechanical analyses of the tango walking (Laguna, 2020), have not been extended to include the metabolic counterpart to mechanics, as generally involved in many other gait studies (Saibene & Minetti, 2003).

Energetic-efficiency studies of locomotion require the determination of the energy cost and a detailed analysis of the mechanical work performed (Saibene & Minetti, 2003; Peyré-Tartaruga et al., 2021). Changes in both aspects can be associated with changes in spatio-temporal variables, for example stride length, stride time and the angular values of lower limb joints during the different phases of the gait cycle (Stenum & Choi, 2020) which in turn can be associated with changes in the knee such as those mentioned above. Besides, walking net metabolic energy can be described as the oxygen cost of transport per unit distance (CoT) (Saibene & Minetti, 2003). Ratio between mechanical work and energy cost is the apparent efficiency of locomotion (*η*) (Saibene & Minetti, 2003). This being an important functional measure, since economy is one of the objectives of walking (Saibene & Minetti, 2003). In addition, exercise economy is known to be influenced by an interaction of physiological, biomechanical, and anthropometric factors (Lundby et al., 2017). From a mechanical perspective, *η* is influenced by the exchange of kinetic and potential energy at the level of the body center of mass (Minetti & Saibene, 1992; Saibene & Minetti, 2003). During walking, these energy components enable a pendulum-like mechanism that reduces the mechanical work required to move the body (Cavagna & Kaneko, 1977). This mechanism can be quantified using indicators such as energy recovery or instantaneous energy recovery (Rinst) which reflects the effectiveness of this exchange, and mechanical energy congruence (%Cong), which describes the temporal coordination between energy fluctuations within the gait cycle (Cavagna et al., 2002; Saibene & Minetti, 2003). Therefore, analyzing these variables may help to elucidate whether tango walking alters the underlying mechanical efficiency of gait.

Biological anthropologists have emphasized how cultural, environmental, and physiological factors interact in shaping variation in human body movement patterns (Willems et al., 2017; Wallace et al., 2018; Lieberman et al., 2020), while biomechanics researchers or comparative physiologists have only recently begun to explicitly address these interactions. Emerging frameworks integrating neuromechanical and sociocultural perspectives, such as the concept of ethnokinesiology, highlight how movement patterns are shaped by the interaction between sensorimotor processes and ecological (including cultural) constraints as dance (Ting et al., 2024). However, locomotion performed for aesthetic purposes rather than functional transport is unlikely to prioritize metabolic efficiency, while favoring instead stylistic and expressive movement patterns, in human as well as in other species (*e.g.*, Western Greebes running on water, quoted in Minetti et al. (2012).

Despite the growing use of tango-based interventions in clinical and rehabilitation contexts, the biomechanical and energetic demands of tango walking remain largely undocumented. This gap is relevant because understanding these demands is essential to begin determining whether tango walking imposes specific locomotor challenges that may underlie its reported therapeutic benefits. This study aims to analyze CoT, mechanical work and different measures associated with the exchange of mechanical energy at the body center of mass level, jointly with spatio-temporal measurements during tango walking in the role of leader. We hypothesized that the aesthetic constraints imposed by tango walking, while controlling for overground speed, would: (I) increase CoT and reduce *η* compared to self-selected walking without musical cueing; and (II) be associated with a coordinated set of modifications in 3D center of mass trajectories, mechanical work variables, and spatiotemporal gait parameters. From a rehabilitation standpoint, specific biomechanical features of tango walking may be operationalized as targeted stimuli to enhance balance control, coordination, and muscular engagement, thereby providing a mechanistic basis for its use in rehabilitation. Quantifying the metabolic and mechanical demands of tango walking among individuals without neurologic deficits who are trained, traditionally, in the dance technique, may elucidate potential mechanisms of action associated with the adapted forms of this technique that have emerged as promising therapeutic modalities.

## Materials & Methods

### Participants

Seventeen advanced tango dancers (nine women and eight men, age 50.4 ± 14.3 yrs, mass 73.1 ± 19.5 kg and stature 1.69 ± 0.09 m) participated in the study. All participants were active dancers with more than 10 years of tango practice and an average frequency of three practices per week. All the participants signed a written informed consent according to the Declaration of Helsinki. The project was approved by the Ethics Committee of the Centro Universitario Regional Litoral Norte, Universidad de la República (Exp. No 311170-000033-22).

### Data collection

During a single session, subjects performed two conditions: 5 min of self-selected walking without music, and 5 min of walking with tango salon technique in the leader role, while they listened to the milonga “La Puñalada”, composed by Horacio A. Castellanos in 1937. The no-music condition was included to obtain a baseline measure of self-selected walking without external rhythmic constraints, allowing comparison with tango walking performed under musical guidance. The musical piece was selected *a priori* because the musical tactus (step frequency 1.98 Hz, or 119 bpm) allowed the technique to be performed with an average speed and stride frequency similar to self-selected walking speed as defined in neurotypical populations (Winner et al., 2024). This selection aimed to allow tango walking to be performed at a comparable overground speed to self-selected walking, thereby facilitating comparison between conditions under similar locomotor speeds. Tango walking was performed without a follower in order to isolate the leader’s motor pattern and minimize variability associated with interpersonal coordination. Participants in the present study performed traditional tango walking as practiced in social tango, and not the adapted form described by Hackney & McKee (2014).

During the trial, subjects continuously walked back and forth on a corridor (10 m in length, 5 m in width, and 4 m in height). They were instructed not to stop at the end of each section, but to turn with a curve, to maintain their momentum. Conditions were counterbalanced across participants to minimize order effects.

### Kinematics data collection

Kinematics data were recorded using an eight-camera motion capture system at 100 Hz (Vicon Motion System, Oxford, UK), positioned to record 2–3 strides for each trial, in the linear portion of the track. Three trials were recorded within the last two minutes of data collection. Eighteen reflective markers were placed on both sides of the body for defining 11 segments and reconstructing their 3D movements (Minetti, Ardigo & Saibene, 1993; Pavei et al., 2017). Filtered marker positions were exported from Vicon’s Nexus 2.11 software (fourth-order zero-lag Butterworth low-pass filter with a cutoff frequency of 6 Hz).

### VO2 data collection

To determine CoT, rates of oxygen consumption and carbon dioxide production were measured with a wearable metabolic system (K5, Cosmed, Italy) for five minutes of quiet upright standing before walking and during the two walking conditions. The K5 metabolic system was calibrated according to the company’s instructions before each session. After wearing the system, each volunteer was instructed to breathe normally and not to speak during the acquisitions. Physiological parameters like hearth rate (HR), oxygen uptake (V̇O2) and respiratory exchange ratio (RER) were monitored during the session by means of a tablet connected to the device *via* Bluetooth. In the time interval between the two test conditions, we ensured that the physiological parameters returned to their upright standing values

### Data processing and analysis

Data processing described in this paper has been performed by custom programs written in Matlab R2022a (The MathWorks, Natick, MA, USA) and Python (Yelós et al., 2026).

### Kinematics data processing and analysis

Center of mass trajectories were reconstructed from kinematics, using Dempster anthropometric tables (Pavei et al., 2017). Owing to the importance of accurately locating the center of mass from kinematic data, we developed a spatial model considering the recommendation of Pavei et al. (2017).

The trajectory and velocity of the heel and 5th-metatarsal markers were used to identify the heel-strike and toe-off events (Pequera, Yelós & Biancardi, 2023). Gait cycles (strides) were cut at the right heel-strike. Variables were calculated as described below and averaged across them for each subject and condition.

Kinematic data from five participants were excluded due to technical issues during motion capture acquisition (*e.g.*, marker loss or incomplete stride reconstruction). As these exclusions were not associated with participant characteristics or experimental conditions, they were considered random and unlikely to introduce systematic bias. All spatio-temporal and energetic variable analyses were therefore conducted on the remaining participants (*n* = 12) using a within-subject design.

The spatio-temporal variables descriptive of the gait were:

• Mean velocity of advance (v); average value of the center of mass cycle speed in the forward direction.
• Stride length (Ls) and stride time (Ts): longitudinal distance in m and time in s from right heel-strike to the subsequent heel-strike.
• Stride frequency (Fs): 1/Ts (Hz).
• Duty factor: fraction of a stride where the foot is on the ground.
• Knee joint kinematics were included as predefined variables to complement the interpretation of center of mass movement and mechanical energy changes, rather than as exploratory outcomes. The time course of the flexion-extension Euler knee joint angle has been computed from the kinematic data of the right thigh and shank segments.
• The 3D contour of the center of mass during one stride (Lissajous curve) was obtained by applying Fourier Analysis to the raw center of mass trajectories reconstructed from kinematics, as described by Minetti, Cisotti & Mian (2011).

The mechanical variables were:

• External mechanical work (Wext), needed to raise and accelerate the center of mass. Determined as the sum of the positive variations of center of mass total mechanical energy curve as explained in Cavagna & Kaneko (1977).
• Kinetic internal work (Wint) (Minetti, Moorhead & Pavei, 2020), was estimated as the sum of the increments of kinetic energy of each limb and of the head and trunk respect to Center of mass in the three planes of movement through the locomotion cycle. Energy transfer between the segments of the same limb was assumed.
• Total mechanical work (Wtot), sum of Wext and Wint (Cavagna & Kaneko, 1977).
• Energy recovery (Rec), determined as:
  (1) \begin{eqnarray*}Rec= \frac{Wv+Wh-Wext}{Wv+Wh} 100\end{eqnarray*}

where Wh is the horizontal external mechanical work, Wv is the vertical work. These work components correspond to homologous energy variations. Horizontal energy (Eh) corresponds to sum of the kinetic energies of the center of mass along the sagittal and lateral axes; vertical energy (Ev), sum of the vertical component of kinetic energy and potential energy of the center of mass. Total external energy of the center of mass (Ext), corresponds to sum of Eh + Ev (Cavagna & Kaneko, 1977; Saibene & Minetti, 2003). In this way the Wext is calculated from external energy variation.

• Mechanical energy congruence percentage (%Cong) was calculated as the number of frames in which d(Eh)/dt and d(Ev)/dt have opposite sign divided by the total number of frames in a gait cycle × 100% (Cavagna et al., 2002; Saibene & Minetti, 2003).
• Instantaneous recovery of mechanical energy (Rinst) was calculated from the absolute value of the changes in Wh, Wv and Wext over the step period, following the approach described by Cavagna et al. (2002). The time course of Rinst was computed continuously over the gait cycle and, when required for analysis, resampled over a normalized step cycle.

### VO2 data processing and analysis

The V̇O2 data of the last minute of each trial was visually inspected to ensure metabolic steady state. Then, the metabolic variables V̇O2 and respiratory exchange ratio (RER) were computed averaging the last-minute range. The upright resting oxygen uptake, representing the baseline of V̇O_2_ (mlO_2_ kg^−1^ min^−1^), was recorded for 5 min at the beginning of the experimental session and computed averaging the last 2 min. Although a 5-min duration is commonly used in gait studies, it is acknowledged that the time required to reach steady state may vary across individuals and conditions (Selinger & Donelan, 2014). Net oxygen uptake was computed by subtracting the baseline of V̇O_2_ from the average V̇O_2_ measured during the last minute of exercise for each walking condition. The net V̇O_2_ was multiplied for the RER caloric equivalent (J mlO_2_^−1^) (Cooke, 2008), to convert the mlO_2_ to Joules. The result was then divided by 60 (s min^−1^). After cancellations, this result is the metabolic power (MetP) in J kg^−1^ s^−1^ or W kg^−1^.

CoT, was then calculated by dividing the MetP by v:

(2) \begin{eqnarray*}CoT= \frac{MetP}{v} \end{eqnarray*}

The locomotion efficiency (*η*) was determined as the ratio between Wtot and CoT.

### Discrete data statistics

Descriptive data are presented as mean ± standard deviation. Normality was assessed using the Shapiro–Wilk test. Differences between conditions were evaluated using paired-samples *t*-tests; when the assumption of normality was violated, the non-parametric equivalent (Wilcoxon signed-rank test) was applied. Within-subjects variability was quantified using the coefficient of variation (CV), defined as the ratio between the standard deviation and the mean, expressed as a percentage. Effect sizes were calculated using Cohen’s d and interpreted as small (≈0.2), medium (≈0.5), and large (≈0.8) (Cohen, 2013).

Discrete statistical analyses were conducted in R (v4.5.2), with the level of significance set at α = 0.05. To control for Type I error due to multiple comparisons, Holm–Bonferroni corrections were applied within conceptually related families of variables (mechanical work, metabolic cost).

To explore the potential influence of stride frequency, additional analyses were performed examining the relationship between individual changes in stride frequency and changes in biomechanical and metabolic variables. For each participant, between-condition differences were computed, and associations were assessed using correlation and simple linear regression.

### Continuous data statistics

SPM1D is a Python library for analyzing and comparing time series data by means of Statistical Parametric Mapping (SPM) (Pataky, 2010), The time course of the flexion-extension knee joint angles of walking strides and of tango walking strides were compared using paired SPM{t} test (two-tailed, alpha = 0.05). The null hypothesis was rejected if SPM{t} statistics exceeded the critical SPM{t} value at least once during the time course of the stride.

The 3D center of mass trajectories were analyzed both as 1D axial components, where each curve represented the center of mass displacements along the x, y or z axes during one stride, and as 3D center of mass trajectory (Luciano et al., 2022). Separate axial center of mass trajectories of walking and tango walking was compared, as above, using two-sample SPM{t} test (two-tailed, alpha = 0.05). The null hypothesis was rejected if SPM{t} statistics exceeded the critical SPM{t} value at least once during the time course of the stride. Mean 3D center of mass trajectories of walking and tango walking was tested using Hotelling’s SPM{T2} statistics with alpha set to 0.05 (Luciano et al., 2022). The null hypothesis was rejected if SPM{T2} statistics exceeded the critical SPM{T2} value at least once during the time course of the stride.

All series were normalized over 0–100% of the gait cycle before analysis. A custom Python (v 3.11) script was built accordingly, using SPM1D, Numpy, Pandas and Matplotlib libraries (Pataky, 2010).

## Results

As expected from the study design, walking speed did not differ between conditions. This suggests that subsequent comparisons primarily reflect differences in gait mechanics and energetics under comparable locomotor demands, although the potential contributions of gait frequency-related constraints cannot be fully excluded.

Results of the spatio-temporal variables (except knee angle) are shown in Table 1. The *t*-test comparisons did only show significant differences between walking and tango walking in duty factor, with a moderate effect size. The CV values were greater in tango walking than in walking, with greatest differences in stride time and stride frequency (Table 1).

**Table 1:** Spatio-temporal gait variables in each walking condition. Data are presented as mean ± SD (CV); *p*-values for paired *t* test analyses and Cohen’s d (effect size); bold values indicate statistically significant *p*-values.

**Condition**	**Speed (m/s)**	**Stride length (m)**	**Stride time (s)**	**Stride frequency (s** ^−1^ **)**	**Duty factor**
**Walking**	1.01 ± 0.17 (16.8%)	1.24 ± 0.10 (8.0%)	1.15 ± 0.09 (7.8%)	0.87 ± 0.06 (6.9%)	0.56 ± 0.02 (3.6%)
**Tango walking**	1.04 ± 0.20 (19.2%)	1.36 ± 0.16 (11.8%)	1.31 ± 0.37 (28.2%)	0.82 ± 0.22 (26.8%)	0.52 ± 0.05 (9.6%)
***p***	0.61	0.066	0.172	0.422	**0.014**
					
**d.f.**	16	11	11	11	11
**Cohen’s d**	0.15	0.89	0.35	0.31	0.63

**Notes.**

d.f. = degrees of freedom (1–n; n = sample size).

Energetics results are displayed in Table 2. It is worth highlighting the variability displayed by most of the energetics variables. In particular, Wv, Wint, energy recovery, Rinst and %Cong showed higher CV in tango walking compared to walking (Table 2).

**Table 2:** Variables associated with CoM energy recovery and mechanical work. Data are presented as mean value ± SD (CV); Holm adjusted p-values and Cohen’s d (effect size). Bold p-values indicate the variables with significant differences.

**Variable**	**Walking**	**Tango walking**	**Holm-adj. *p* value**	**d.f.**	**Cohen’s d**
**Wv (JKg** ^−1^ **m** ^−1^ **)**	0.46 ± 0.11 (23.9%)	0.35 ± 0.20 (57.1%)	0.238	11	0.62
**Wh (JKg** ^−1^ **m** ^−1^ **)**	0.28 ± 0.06 (21.4%)	0.39 ± 0.08 (20.5%)	0.072	11	1.52
**Wext (JKg** ^−1^ **m** ^−1^ **)**	0.34 ± 0.12 (35.3%)	0.36 ± 0.09 (25.0%)	1	11	0.18
**Wint (JKg** ^−1^ **m** ^−1^ **)**	0.24 ± 0.04 (16.7%)	0.21 ± 0.08 (38.1%)	1	11	0.42
**Wtot (JKg** ^−1^ **m** ^−1^ **)**	0.58 ± 0.11 (19.0%)	0.57 ± 0.09 (15.8%)	1	11	0.08
**En. recovery (%)**	55.1 ± 11.0 (20.0%)	49.1 ± 12.9 (26.3%)	1	11	0.50
**Rinst (%)**	48.2 ± 4.9 (10.2%)	48.6 ± 9.5 (19.5%)	1	11	0.05
**%Cong (%)**	23.4 ± 6.2 (26.5%)	25.5 ± 12.6 (49.4%)	1	11	0.21
**MetP (WKg** ^−1^ **)**	1.89 ± 0.65 (34.4%)	2.94 ± 0.81 (27.5%)	**0.001**	16	1.43
**CoT (JKg** ^−1^ **m** ^−1^ **)**	1.95 ± 0.52 (26.7%)	2.92 ± 0.93 (31.8%)	**0.011**	16	1.03
*η*	0.452 ± 0.171 (37.8%)	0.213 ± 0.090 (42.2%)	**0.0012**	11	1.75

**Notes.**

d.f. = degrees of freedom (1–n; n = sample size).

Significant differences were observed in CoT, MetP and *η*, with higher CoT and MetP and lower *η* in tango walking compared to walking (Table 2). Changes in stride frequency were moderately associated with variations in several variables, including CoT (*R*^2^ = 0.33) and *η* (*R*^2^ = 0.26), while virtually no proportion of the variance was explained for metabolic power (*R*^2^ = 0.004).

**Figure 1:**
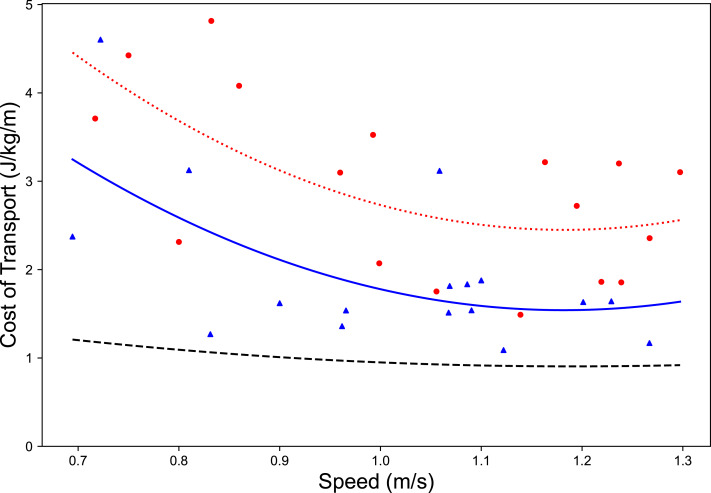
Overall Cost of Transport *vs* Speed. Triangles: walking, fitted by continuous curve (Equation: CoT = 7.18V^2^−16.98v + 11.58, v = speed, R^2^ = 0.367); Circles: tango walking, fitted by dotted curve (Equation: CoT = 8.42v^2^−19.91v + 14.22, v = speed, R^2^ = 0.377). Each point represents the CoT of one subject. The difference between the two equation is plotted as black dashed line (see (3)).

The CoT trend *versus* speed is shown in Fig. 1, where two quadratic equations were fitted to the experimental points. The difference between the two polynomial equations gave the following new equation, also plotted in Fig. 1, which will be subsequently discussed:

(3) \begin{eqnarray*}CoT=1.23{v}^{2}-2.93v+2.65\end{eqnarray*}

Walking one meter under tango conditions was associated with an approximately 1 J kg^−1^ higher cost compared to walk (Fig. 1).

**Figure 2:**
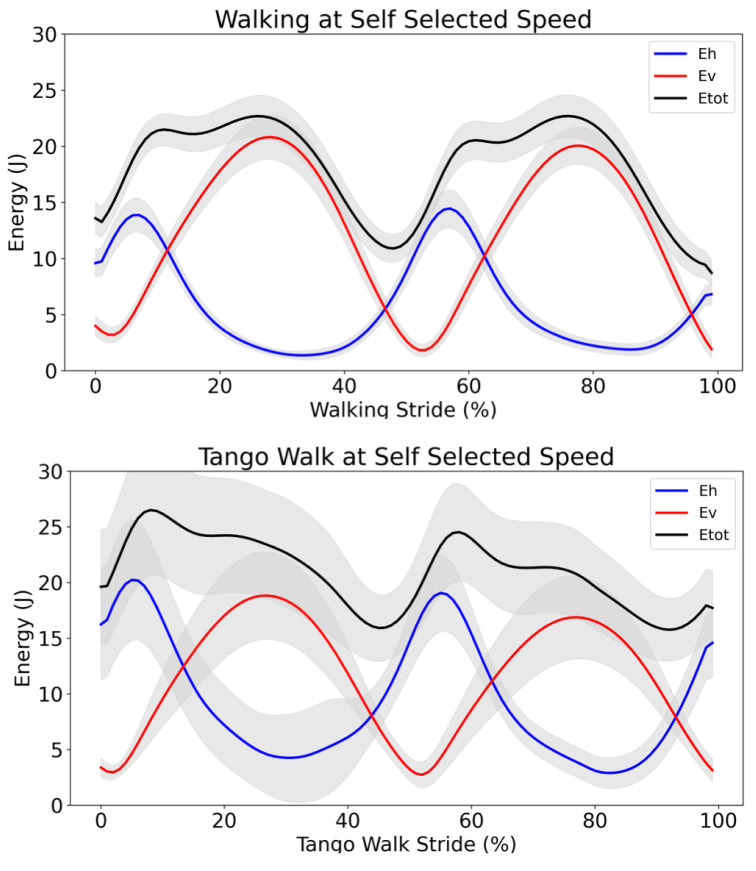
Mean time course of CoM energies during one stride (0 to 100% of a walking cycle). Eh, Horizontal energy; Ev, Vertical energy; Etot, Total mechanical energy (sum of Eh and Ev); 0% and 100% correspond to the right heel strike; Curves correspond to the average value of 12 subjects; Grey areas correspond to 95% confidence interval.

Differences in Wv and Wh were not statistically significant after Holm-Bonferroni correction for multiple comparisons; however, their medium to large effect size indicate a biomechanical trend consistent with the patterns shown in Fig. 2, where the average time course of energies associated with the center of mass during the stride of both gaits is plotted against time. The center of mass energies of tango walking displayed more variability, as shown by the larger confidence interval areas, but average fluctuations of Eh were larger than in walking. Conversely, Ev of walking presented a larger range of values compared to tango walking.

**Figure 3:**
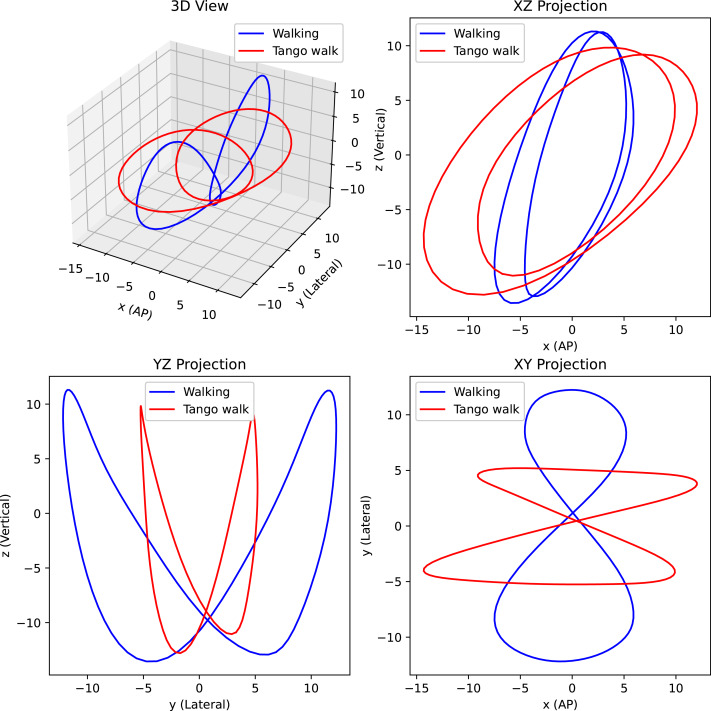
Comparison of the 3D CoM trajectories over a normalized stride cycle. The blue Lissajous curve corresponds to walking, while the red refers to tango walking. Projections on the three coordinate planes are provided: X (AP): Anteroposterior axis; Y (lateral): Mediolateral axis; Z (vertical): Vertical axis; (units in mm). Curves correspond to the average value of 56 representative strides.

The differences between vertical and horizontal center of mass displacements during walking and tango walking are also evident in the next Fig. 3, where the averaged 3D center of mass Lissajous trajectories of walking and tango walking are shown, along with their projection on the coordinate planes.

**Figure 4:**
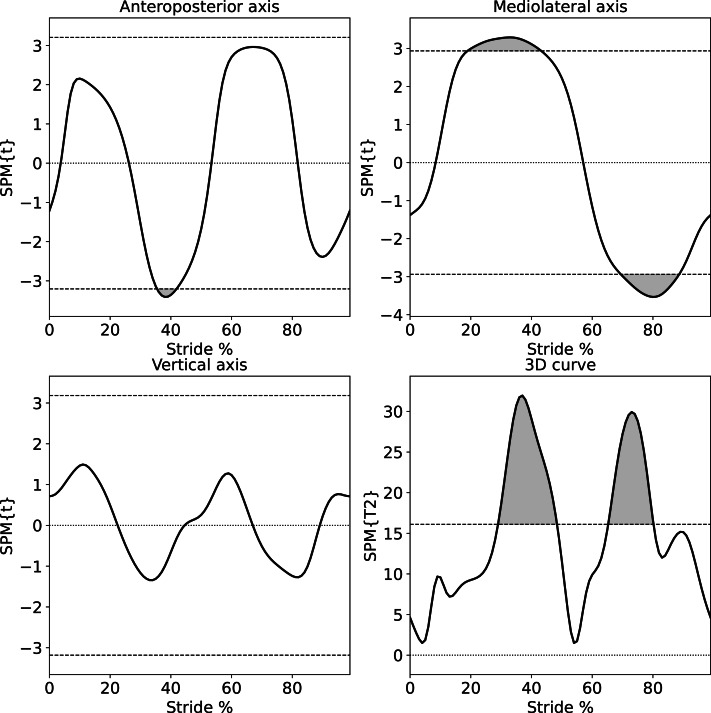
Statistical parametric mapping comparison between walking and tango walking Lissajous curves. 0% and 100% correspond to the right heel strike. Reference alpha values are reported and marked as dotted lines. Significant differences are identified by shaded areas. SPMt: Values of two samples *t* statistics; SPMT2: values of Hotelling’s statistics.

The critical SPM threshold was exceeded on the anteroposterior (*t* = 3.21, *p* = 0.023) and mediolateral axis (*t* = 2.94, *p* = 0.0039), revealing significant differences in the trajectory of the center of mass between tango walking and walking in these axes (Fig. 4). The critical SPM threshold was also exceeded in the 3D center of mass trajectory test (T2 = 16.1, *p* < 0.001). In both 1D and 3D analyses, the walking and tango walking curves diverged more during the midstance phases (right and left).

**Figure 5:**
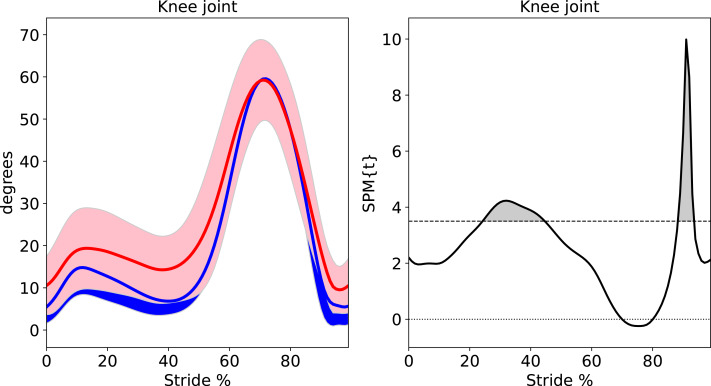
Knee joint angle comparison. Left pane: Trajectories of the right knee joint flexion-extension angle averaged during one stride of walking (blue curves) and tango walking (red curves): 0% and 100% correspond to the right heel strike. Curves correspond to the average value of 12 subjects. Right pane: Statistical Parametric Mapping comparison between walking and tango walking strides (two-sample *t*-test, two tailed). Reference alpha values are reported and marked as dotted lines. Significant differences are identified by shaded areas; SPMt: Values of paired *t* statistics.

As expected based on tango technique, knee flexion during stance was consistently greater in tango walking compared to self-selected walking. Importantly, the present analysis provides a quantitative characterization of this pattern across the gait cycle, revealing that differences were not uniform but concentrated in specific phases of stance, as identified by SPM (Fig. 5).

The curves appeared to diverge more at the beginning of the cycle, from heel-strike until about 60% of the stride, during the right stance, and just before the following heel-strike, at the end of the right swing phase (*t* = 3.50, *p* = 0.026). A spreadsheet with detailed results is available as Supplementary File.

## Discussion

This work represents the first biomechanical description of tango gait in which energetic aspects are evaluated alongside spatiotemporal variables, allowing a direct comparison between self-selected walking and tango walking as an aesthetic form of locomotion. We hypothesized that, while controlling overground speed, tango walking would (i) increase the CoT and reduce *η* relative to self-selected walking without musical cueing, and (ii) be associated with specific modifications in center of mass trajectories, mechanical work variables, and spatiotemporal gait parameters. The results support the first hypothesis and reveal a consistent pattern of biomechanical modifications associated with the increased energetic cost of tango walking.

The absence of differences in walking speed between conditions, as ensured by the study design, is crucial for the aspects under discussion, as the variables considered in this work are reliant on speed (Saibene & Minetti, 2003). If there were significant differences in speed it would not be feasible to discuss whether changes in other variables are linked to changes in the technique. Therefore, the deliberate choice of music becomes a crucial consideration when utilizing tango as a rehabilitation strategy. The music chosen for this study corresponds to a milonga, a fast-paced rhythm within tango (Selles, 2004).

The average values and CV obtained in this study during walking were within the range of values previously cited for stride length (Rock et al., 2018; Torres et al., 2024), stride time (Rock et al., 2018; Torres et al., 2024), stride frequency (Minetti et al., 1995; Torres et al., 2024), and duty factor (Nardello, Ardigò & Minetti, 2011; Torres et al., 2024). Our findings for spatiotemporal variables in tango walking do not align with the subjective observations reported by dancers in Laguna (2020). These dancers pointed out that when walking in tango mode, the automatic mechanisms governing human gait are lost due to the expressive intentionality of the dance. Laguna (2020), indicates that by transforming the street step into a tango step, a significant reduction of steps per minute is produced, and the individual step distance is approximately 20% greater than in street mode. It is possible that the observations made by Laguna, (2020), were associated with changes in speed, since this variable was not controlled.

It is important to highlight that, although it did not differ significantly at the group level, substantial inter-individual variability was observed for stride frequency, energy recovery, Wint, Wv. Additional analyses indicated that stride frequency changes were associated with variations in several variables, including energy recovery and CoT. However, the relatively low explanatory power for other variables, particularly metabolic power and congruency, suggests that tango-specific biomechanical adaptations may also play a role beyond changes in stride frequency. Therefore, the observed changes which we will discuss later cannot be attributed solely to stride frequency differences, and at least in part are due to the tango walking technique.

Previous work has shown that, at a given walking speed, humans tend to select a step frequency that minimizes energy expenditure (Minetti et al., 1995). However, in our work under tango walking conditions, CoT and *η* were significantly increased despite similar walking speeds and stride frequency. In human walking, net CoT exhibits a U-shaped relationship with speed, with a minimum of about 2 J kg^−1^m^−1^ at speeds ranging from 1.1 to 1.4 m s^−1^ (Saibene & Minetti, 2003; Mian et al., 2006; Peyré-Tartaruga et al., 2021). So, the CoT values recorded for the self-selected speed (1.95 ± 0.90 J kg^−1^ m^−1^) fall within the expected range for the speed developed in this study (1.015 ± 0.17 m s^−1^). Meanwhile, when walking with tango technique, the CoT significantly increased about 50% (2.925 ± 0.98 J kg^−1^m^−1^, Table 2), reinforcing the idea that tango walking imposes additional energetic demands without a proportional increase in forward velocity. Although both gaits displayed similar distribution of CoT values *vs.* speed, with higher CoT at lower speeds, and a trend toward energetically “optimal” speeds around 1.1 to 1.4 m s^−1^ (Fig. 1) (Saibene & Minetti, 2003; Mian et al., 2006; Peyré-Tartaruga et al., 2021). The CoT of tango walking was about halfway between the optimum for walking and that obtained for CoT in running (4 J kg^−1^m^−1^) (Saibene & Minetti, 2003; Peyré-Tartaruga et al., 2021; Biancardi et al., 2023), and similar to values observed in populations with altered motor control or coordination (Bona et al., 2017; Mariman et al., 2022). The difference in CoT between walking conditions was also comparable to that reported between young and older adults (Mian et al., 2006), suggesting that tango walking may induce a locomotor pattern with reduced energetic efficiency analogous to that observed in less optimized gait strategies. The concurrent increase in MetP and reduced efficiency in the conversion of metabolic energy into forward mechanical work, consistent with the interpretation that tango walking involves less economical neuromuscular strategies.

Our mechanical results for walking are consistent with values reported in the literature for Wext, Wint, and Wtot in healthy adults at self-selected speeds (Minetti et al., 1995; Saibene & Minetti, 2003; Bona et al., 2017; Torres et al., 2024), as well as for recovery-related metrics such as energy recovery, Rinst, and %Cong (Gomeñuka et al., 2016; Bona et al., 2017; Torres et al., 2024). These variables collectively describe the mechanical demands of gait and the efficiency of pendular energy exchange.

In many altered locomotor patterns, increases in mechanical work (Wext, Wint, Wtot) are typically associated with higher metabolic demand (Bona et al., 2017; Peyré-Tartaruga & Coertjens, 2018), highlighting the multifactorial nature of locomotor economy (Lundby et al., 2017). Similarly, reductions in pendular recovery have been linked to increased CoT (Bona et al., 2017). Therefore, it was expected that variations in mechanical variables would be associated with the changes in CoT observed in tango walking. However, the mechanical variables showed no differences (Table 2), suggesting that the increase in CoT and MetP are not primarily associated with changes in external mechanical output. However, the implications of the trends detected in Wv and Wh (Eq. (1), Table 2), will be discussed shortly.

Greater dispersion of data was observed during tango walking in both discrete and continuous variables, including the time course of center of mass energies (Fig. 2) and the center of mass trajectory (Fig. 3). While discrete mechanical variables showed greater dispersion, with reduced Wv and higher Wh, continuous analyses provided further insight into when these alterations occurred during the gait cycle. SPM and the reconstructed 3D center of mass trajectory (Figs. 3 and 4) showed that during single support phases, the center of mass trajectory in tango walking significantly diverged from that of normal walking, with a marked reduction in lateral oscillations. Changes in center of mass trajectory are indicative of modifications in movement organization, suggesting that tango walking may constrain mediolateral motion, likely through a narrower step width. Such a walking style, often observed in contexts emphasizing aesthetic control, supports the interpretation of tango walking as a locomotor pattern that increases metabolic cost without a corresponding increase in Wext or Wint, but it could affect, even if only minimally, Wv and Wh.

The analysis of the time course of knee flexion–extension (Fig. 5) confirms a key characteristic of tango walking that is explicitly emphasized in its pedagogy. However, the present study extends this qualitative understanding by providing a quantitative characterization of its magnitude and temporal distribution across the gait cycle. During tango walking, trained tango dancers exhibited significantly greater knee flexion throughout stance. Importantly, the present results provide, to our knowledge, the first objective characterization of the temporal structure and magnitude of this feature across the gait cycle. This kinematic pattern is consistent with a more flexed posture, which is associated with the reduced vertical excursion of the center of mass, and results observed in Figs. 2–4. Walking with increased knee flexion during stance has been associated with higher joint moments at the knee and ankle (Gordon, Ferris & Kuo, 2009), reflecting a greater requirement for active muscle force to support body weight and control limb motion. In addition, the reduced contact time (duty factor; Table 1) may further increase the rate of force production, contributing to greater metabolic demand of the lower limb musculature (McMahon, Valiant & Frederick, 1987; Gordon, Ferris & Kuo, 2009; Beck et al., 2020). In this context, the additional CoT observed during tango walking (≈1 J kg^−1^ m^−1^; Table 2, Fig. 1) may partly reflect increased muscular activity associated with postural control and joint stabilization. Rather than resulting from increased external mechanical work, these findings support the interpretation that tango walking involves a less efficient neuromuscular strategy, with greater reliance on active control mechanisms. Increased metabolic CoT in the absence of substantial changes in external mechanical work may also be associated with greater muscle activation for postural stabilization and joint control. In particular, co-contraction strategies have been shown to increase metabolic demand while having limited effects on mechanical work output (Minetti, Pinkerton & Zamparo, 2001; Minetti & Pavei, 2018). Although electromyographic data were not collected in the present study, this mechanism may contribute to the increased CoT observed during tango walking.

In this way, according to the ethnokinesiology framework proposed by Ting et al. (2024), rather than representing a simple biomechanical variation of normal walking. This perspective may help explain why tango walking increases metabolic cost without clear changes in mechanical work, as movement execution may prioritize aesthetic and expressive constraints over energetic efficiency.

In summary, tango walking emerges as a distinct locomotor pattern characterized by increased CoT and MetP, reduced *η* and coordinated spatial adaptations under aesthetic and rhythmic constraints. From an applied perspective, the increased metabolic demand during tango walking suggests its potential as a moderate-intensity motor task that may complement traditional exercise approaches. On the other hand, it is relevant to consider that different aspects of gait modulation may place distinct demands on motor and cognitive systems. Recent work on dance-based gait modifications (Rosenberg et al., 2023) has shown that spatial components of movement (*e.g.*, joint kinematics and movement amplitude), such as those predominantly observed in the present study, are more closely associated with motor capacities such as strength and balance, whereas temporal components (*e.g.*, step timing and rhythmic sequencing) rely more heavily on cognitive processes. In this context, the tango walking pattern analyzed here may primarily represent a motor challenge driven by spatial reorganization of gait, while still incorporating rhythmic constraints that could engage cognitive processes. This distinction may be particularly relevant when considering the design of tango-based interventions for populations with motor and/or cognitive impairments.

A limitation of the present study is the relatively small sample size, which may restrict the generalizability of the findings and the stability of the analyses. In addition, the exploratory nature of the association analyses, together with the limited sample size, precludes strong inferences regarding causal relationships between biomechanical variables and metabolic cost.

Kinematic data were unavailable for a subset of participants due to marker loss. Although this was not related to participant characteristics or experimental conditions and was therefore considered random and unlikely to introduce systematic bias, the results should nevertheless be interpreted with caution.

Tango walking was examined exclusively in the leader role and without a partner. While this approach allowed isolation of a fundamental motor component of tango walking, it does not capture the additional biomechanical and coordinative demands associated with interpersonal interaction, which may influence gait dynamics in real-world or therapeutic contexts.

Moreover, only one tango subgenre (milonga) was assessed, limiting conclusions regarding the broader tango repertoire. The use of a fixed musical tactus, together with the absence of a frequency-matched control condition, further limits the ability to fully dissociate the effects of stride frequency from tango-specific gait adaptations.

In addition, although steady-state oxygen uptake was visually confirmed in all trials, the time required to reach metabolic steady state may vary across individuals and conditions, and a fixed trial duration may not fully capture this variability (Selinger & Donelan, 2014).

Finally, it should be noted that the present study examined traditional tango walking with experienced dancers rather than adapted tango protocols used in rehabilitation. Therefore, caution is warranted when extrapolating these findings to clinical populations, where movement patterns may be intentionally modified for safety and therapeutic purposes.

Future research should investigate larger and more diverse cohorts, including novice and clinical populations, to determine the reproducibility of the findings. The inclusion of electromyographic analyses would clarify the role of muscle co-contractions in the increased metabolic cost. Comparative studies of leader and follower roles, as well as different tango subgenres, will be essential to fully characterize the biomechanical and energetic demands of tango dance and to optimize its use in rehabilitation settings.

## Conclusions

Tango walking performed in the leader role at milonga rhythm, while maintaining comparable overground speed to self-selected walking, is characterized by a substantial increase in metabolic cost without proportional changes in total mechanical work. This pattern is accompanied by specific spatial biomechanical adaptations, including greater knee flexion during stance and modifications in center-of-mass trajectory, particularly reduced mediolateral oscillations.

These findings support the interpretation of tango walking as a distinct locomotor pattern shaped by aesthetic constraints, involving a reorganization of motor behavior associated with increased energetic demand.

While the present results provide a quantitative basis for understanding the physical demands of tango walking, these findings may help inform future applications in rehabilitation contexts. However, further research is required to clarify the underlying neuromuscular mechanisms and their relevance in clinical populations.

## Supplemental Information

10.7717/peerj.21581/supp-1 Supplemental Information 1 Detailed results per subject and gait Id: subject Id; Gait: walk or tango-walk, both at the self-selected speed.Includes 15 variables (spatio-temporal, mechanics and metabolics)

10.7717/peerj.21581/supp-2 Supplemental Information 2 Matlab (R24B) code for processing kinematic data Zipfile includes:
Matlab script (Mechanics_tango)
Required data (mass.csv): mass of the subjects
Dataset: 67 csv files coded as follow: ”sX”, where “X” is an integer: Identifies the subject number “tango” or “vas”: Identify the gait (tango = tango walk; vas = walking) N (progressive number 1 to 3): Three clips were recorded for each trial. This progressive number identifies the clip or record number.
This Dataset is also available in a public repository at https://doi.org/10.60895/redata/6Q1BD8

10.7717/peerj.21581/supp-3 Supplemental Information 3 Statistical Parametric Mapping, knee angles Zipfie includes:
Python scripts Angles_SPM.py
Dataset of knee angles (2 csv files)
Run the script with dataset in the same directory for obtaining the SPM paired-t results and figures

10.7717/peerj.21581/supp-4 Supplemental Information 4 Python script for obtaining Lissajous curves Zipfile includes:
Python script Lissajous.py: obtain lissajous curves of the CM trajectoriesInput csv files: single strides 3D CM position. Files obtained from the Matlab processing of kinematic trials.

10.7717/peerj.21581/supp-5 Supplemental Information 5 Statistical Parametric Mapping of CM 3D trajectories Zipfile includes:
Python script Lissaj_SPM.py: perform SPM (2D and 3D) and save figures6 Datasets with paired normalized 1D trajectories (x, y, z; W and TW)

10.7717/peerj.21581/supp-6 Supplemental Information 6 README_DadaDictionary Include a detailed data dictionary for interpreting the raw data and supplementary files.

## Additional Information and Declarations

### Data Availability

The following information was supplied regarding data availability: The code is available in the Supplemental Files. The data is available at Redata: - Torres, Carol; Biancardi, Carlo; Factory, Gabriel, 2025, ”Breath-by-breath cardiometabolic data during walking and tango walk at self-selected speed,” https://doi.org/10.60895/redata/MZJKHA, Uruguay Open Research Data Repository, V1 - Torres, Carol; Minetti, Alberto; Biancardi, Carlo; Fábrica, Gabriel, 2025, “Kinematics raw data of walking and tango walk at self-selected speed”, https://doi.org/10.60895/redata/6Q1BD8, Repositorio de datos abiertos de investigación de Uruguay, V2, UNF:6:g4eaY/QdZXeLbsSISJ09+g== [fileUNF].
