# Supplementary material for: Energetics, kinematics, and physiologic aspects of tango walking in the leader role
Source: PeerJ. 2026 Aug 6;14:e21581. doi: 10.7717/peerj.21581 (PMC13453143; doi:10.7717/peerj.21581)
Supplement: Supplementary file 1 [file peerj-14-21581-s006.docx]

Energetics and physiological aspects of Tango dance walking in the role of leader nergetics, kinematics, and physiologic aspects of tango walking in the leader role

Carol Torres, Alberto E. Minetti, Carlo M. Biancardi, Gabriel Fábrica

Herewith the description of the supplementary table and the research raw data made available.

1) S1 Table

All the results averaged for subject and gait.

Id: subject Id

Gait: Walk or Tango

Body Mass: in Kg

Speed: average speed in m/s

Stride Frequency in Hz

Stride Length in m

Duty_factor: the relative stance time expressed as fraction (1 = the total stride time)

WV (J/kg/m): the vertical work, sum of increments of the vertical energy over a stride.

WH (J/kg/m): the horizontal work, sum of increments of the horizontal energy over a stride.

WEXT (J/kg/m): the external work, sum of increments of the total energy over a stride.

WINT (J/kg/m): the internal work, sum of increments of the translational and rotational energies over a stride.

Recovery: the fraction of mechanical energy recovered by pendular exchange.

InstRec: Instantaneous recovery, the fraction of mechanical energy recovered frame by frame, and averaged.

Congruency: the fraction of a stride when both potential and kinetic energy increments or decrements.

MetPow (W/kg): the metabolic power.

CoT (J/kg/m): the cost of transport.

Wtot (J/kg/m): the total work, sum of WEXT and WINT.

Eff: Apparent efficiency.

2) MatlabCode.zip: Kinematics raw data of walking and tango walk at self-selected speed

Repository ANII - Redata: <https://doi.org/10.60895/redata/6Q1BD8>

Textfiles filenames are coded as follow:

“sX”, where “X” is an integer: Identifies the subject number

“tango” or “vas”: Identify the gait (tango = tango walk; vas = walking)

N (progressive number 1 to 3): Three clips were recorded for each trial. This progressive number identifies the clip or record number.

Each csv file include 56 columns: - Frame and subframe are indices columns - The following columns are grouped by three, and named as right or left (R, L) + the name of the body marker + the axis (e.g. RLOBx, RLOBy, RLOBz, for a total of 9 anatomical positions and 18 markers).

LOB = Temporal lobe SH = Acromion ELB = Elbow WR = Ulnar stiloyd process GT = Great Trochanter KN = Knee (lateral) AN = Malleolus lateral HEE = Heel MT = 5th Metatarsal

The 3D space was calibrated following the right hand rule, with the axis "X" = mediolateral; "Y" = anteroposterior; "Z" = vertical.

The index file "mass.csv" includes the mass (Kg) of each subject, and other info useful for automatic data processing with Python or Matlab.

MatlabCode.zip includes one Matlab script (Mechanics_tango.m) used for processing the files.

3) Lissajous_CM_py.zip

Textfiles filenames are coded as follow:

“sX”, where “X” is an integer: Identifies the subject number

“tango” or “vas”: Identify the gait (tango = tango walk; vas = walking)

N (progressive number 1 to 3): Three clips were recorded for each trial. This progressive number identifies the clip or record number.

“CM”: stands for centre of mass.

“x.xx”: These last four characters store the trial speed in m/s.

Each csv file include 3 columns: cmx; cmy; cmz.

The 3D space was calibrated following the right hand rule, with the axis "X" = mediolateral; "Y" = anteroposterior; "Z" = vertical.

Lissajous.py: python script for processing CM files. It produces and save:

- Normalized CM csv files: normalized to a length of 100 points
- Harmonics csv files: the 10 harmonics of the Fourier analysis
- Lissaj csv files: the normalized Lissajous curve
- Lissajous png files: plot of the Lissajous vs. normalized curves

4) Lissa_SPM.zip

Textfiles filenames are coded as follow: “Lissa_” + axis (x, y, z) + “_” + vas (walk) or tango.

They include 1D arrays of the Lissajous curves for SPM analysis. Each row is a curve. Columns are frames (1 to 100).

Lissaj_SPM.py: python script for processing the included files and obtaining SPM results (textfles) and plots (png files).

5) knee_angles_py.zip

Two csv files (tango and walk). They include 1D arrays of the knee angle values. Each row is a subject. Columns are frames (1 to 100).

Angles_SPM.py: python script for processing the included files and obtaining SPM results (textfles) and plots (png files).
